# *Wolffia globosa*-Based Nutritious Snack Formulation with High Protein and Dietary Fiber Contents

**DOI:** 10.3390/foods12142647

**Published:** 2023-07-09

**Authors:** Nattira On-Nom, Prapatsorn Promdang, Woorawee Inthachat, Panyaporn Kanoongon, Yuraporn Sahasakul, Chaowanee Chupeerach, Uthaiwan Suttisansanee, Piya Temviriyanukul

**Affiliations:** Food and Nutrition Academic and Research Cluster, Institute of Nutrition, Mahidol University, Salaya, Phuttamonthon, Nakhon Pathom 73170, Thailand; nattira.onn@mahidol.ac.th (N.O.-N.);

**Keywords:** dietary fibers, duckweed, food security, food fortification, essential amino acids, healthy snack, protein, genotoxicity, *Wolffia globosa*

## Abstract

*Wolffia globosa* (*W. globosa*) or duckweed is a small aquatic plant with high protein, dietary fiber, and lipid contents that can be combined with food products to develop nutritious snacks as one strategy to mitigate malnutrition. Here, response surface methodology (RSM) with mixture design was used to develop snacks from *W. globosa* freeze-dried powder (WP). The physical properties, proximate analysis, amino acid profiles, sensory evaluation, phytochemical analysis, antioxidant properties, and genotoxicity (Ames test) of the snacks were evaluated. The optimal *W. globosa* snack formula was 64% glutinous rice flour, 10% tapioca flour, and 26% WP, giving a highly desirable liking score of 1.00. Addition of WP increased crude protein, essential amino acids, and dietary fiber compared with the control snack by 51%, 147%, and 83%, respectively. According to the Thai recommended daily intakes, the developed *W. globosa* snack had high protein and dietary fiber. Phytochemical contents and antioxidant activities of the *W. globosa* snack such as total phenolic contents (TPCs), total flavonoid contents (TFCs), ferric ion reducing antioxidant power (FRAP) activity, and oxygen radical absorbance capacity (ORAC) activity were significantly higher than the control snack. The novel combination of WP with snack product ingredients greatly enhanced nutritional value.

## 1. Introduction

The World Health Organization (WHO) states that imbalance of essential macro and micronutrients characterizes malnutrition, which can present as overnutrition or undernutrition. Protein deficiency can lead to kwashiorkor or marasmus [1], while inadequate dietary fiber consumption induces constipation and increases the risk of colon cancer [2]. The WHO advises consuming at least 400 g of fruits and vegetables every day to ameliorate the risks of noncommunicable diseases (NCDs) [3]. The National Health Service (NHS) of the United Kingdom gives primary dietary advice to treat malnutrition including (i) consume fortified meals rich in calories and protein, (ii) intake beverages high in calories, and (iii) snack between meals [4]. Several countries have included healthy snacks in their dietary guidelines. In France, *Le Guide Alimentaire Pour Tous* recommends consuming a regular snack instead of eating mindlessly or snacking continuously. For snack foods, this guide recommends yogurt, milk, fruit, fruit juice, vegetables, or bread with butter or jam, while Switzerland provides an entire page of healthy snack ideas, which includes fruit, vegetables, whole-grain breads, cheese, yogurt, milk, and nuts but advises against sweets and fatty, salty snacks [5]. These recommendations highlight the prospect of using healthy snacks as an appropriate treatment for malnutrition or diseases caused by nutritional imbalances, such as constipation, kwashiorkor, or marasmus, as stated previously.

*Wolffia globosa* (Roxb.) Hartog and Plas (*W. globosa*), commonly called duckweed, belongs to the Lemnaceae family, which covers five genera including *Landoltia*, *Lemna*, *Spirodela*, *Wolffia*, and *Wolffiella*, with variable plant shapes and living conditions. Duckweeds are small (less than 5 mm long), free-floating aquatic plants with a flat oval form but no true leaves and stem [6]. Duckweeds reproduce mostly through asexual budding or vegetative reproduction, and sometimes through sexual reproduction or blooming, resulting in rapidly expanding plants with size doubling time every 2.3 days [7], enabling fast mass production. Recently, duckweeds have attracted interest because of their high protein, dietary fiber, fat, and phytochemical contents. They have been proposed as practical, cost-effective, and innovative sources of macro and micronutrients, with the goal of minimizing environmental problems and boosting food security [8]. The amount of crude protein in duckweeds varies by species. For example, *Lemna* sp. has 16.0% dry weight (DW), *Landoltia* sp. has 20.0–28.7% DW, *Wolffia arrhiza* has 19.8% DW and *W. globosa* has 48.2% [8,9]. The high protein levels found in duckweeds are comparable to pork, beef, and eggs which contain crude protein at 27.7%, 40.5%, and 52.7% DW, respectively [10]. High protein content does not reflect protein quality in terms of nutrition; thus, essential amino acids (EAAs) in duckweeds also need to be characterized. Interestingly, duckweeds contain all the EAAs suggested by WHO guidelines for adults [7,11]. Appenroth et al. found that the average amino acid compositions of isoleucine, histidine, lysine, valine, and leucine in diverse *Wolffia* species were 20–32% higher than the WHO recommendations, whereas threonine was 78% higher [7]. *Wolffia* species have high dietary fiber, carotenoids, zinc, potassium, manganese, and iron, with dietary fiber reported between 10.7 and 14.72% DW [12] and carotenoids including (all-E)-lutein and (all-E)-violaxanthin 30.8 and 46 mg/100 g DW, respectively [13]. Non-nutrient compounds like phytosterols, phenolics and flavonoids including phytol, sitosterol, β-sitosterol, ferulic acid, luteolin7-*O*-β-d-glucoside, and kaempferol have also been reported in *Wolffia* species [7,13,14]. These compounds are well-known for their wide-ranging health benefits covering antioxidant and cholesterol-lowering properties [15,16]. Therefore, duckweeds show promise as a food ingredient with high nutritional health benefits. For instance, anemic rats treated with a low-meat Mediterranean diet supplemented with *W. globosa* showed the restoration of hemoglobin, indicating its efficiency in reversal of anemia [17].

Duckweeds include various macro and micronutrients that promote nutrition but may have a foul odor emanating from protein degradation [18]. The plant’s potential applications are greatly restricted by the fact that only fresh duckweed can be used for cooking. Thus, this study aimed to develop a *W. globosa*-based snack with high protein and dietary fiber content from freeze-dried *W. globosa* powder. Response surface methodology (RSM) was used to develop a sensorily acceptable snack formula that was characterized by physical properties, health-promoting abilities, and genotoxicity. Results suggested that duckweed snacks can be marketed as high in protein and dietary fiber with antioxidant characteristics and genome safety. Duckweed snacks show promise as nutritional future food alternatives.

## 2. Materials and Methods

### 2.1. Raw Materials

Glutinous rice flour (8.8% moisture, 6.6% protein, 0.4% fat, 82.7% carbohydrates) and tapioca flour (12.1% moisture, 0.3% protein, 0.1% fat, 87.2% carbohydrates) were purchased from Bangkok Inter Food Co. Ltd., Bangkok, Thailand. Soybean oil was obtained from Angoon brand, Thai vegetable oil PLC, Bangkok, Thailand. Freeze-dried *W. globosa* powder (WP) was received from an organic farm in Ayutthaya province, Thailand.

### 2.2. Preparation of Snack Product

The control snack formula was modified from Khemthong et al. [19], and consisted of glutinous rice flour (27% *w*/*w*), tapioca flour (7% *w*/*w*), water (62% *w*/*w*) and soybean oil (4% *w*/*w*). All ingredients were mixed under low heat in a pan to swell the starch granules. The dough was formed in a square shape (1 mm thickness) and dried in a hot air oven (electric convection dryer 12 kW/380 V, Kluay Num Thai, Bangkok, Thailand) at 70 °C for 90 min. The semi-dried dough was then cut into 4.5 cm × 5 cm pieces and dried (at 70 °C) again until the moisture content was lower than 8%. The dried dough was kept in an aluminum foil bag at 4 °C until used. For puffing the snack, the dried dough was placed in an electric oven (model Tecno+, The Signature Brand Co., Ltd., Bangkok, Thailand) at 150 °C for 3 min and cooled at room temperature (28 ± 2 °C) before packing in aluminum foil bags for future analysis.

### 2.3. Experimental Design by Response Surface Methodology (RSM)

A mixture design was constructed to optimize the levels of independent variables to develop the high protein snack including glutinous rice flour (GF), tapioca flour (TF) and freeze-dried *W. globosa* powder (WP), and to investigate their effects on the physical properties, nutritional values, and sensory attributes. High and low limits of the three independent variables were set following a preliminary experiment as GF (X_1_) = 50–70% *w*/*w*, TF (X_2_) = 10–30% *w*/*w*, and WP (X_3_) = 20–40% *w*/*w*. The sum of all mixture components added up to 100% *w*/*w*, i.e., GF + TF + WP = 100% *w*/*w*. The other ingredients including soybean oil and water were kept constant. The snack was prepared as described in the previous section. The experimental design consisted of 10 runs, with design levels shown in Table 1. RSM was used to investigate the relationships between the independent and response variables using a regression model. The correlation coefficient of determination (R^2^) and significant *p* value were used to judge the adequacy of model fit. The desirability function tool of RSM was used to generate optimal snack formulation under the criteria of protein >10 g/100 g (10% of the Thai recommended daily intake, Thai RDI) [20,21] and overall liking score more than 6 (like slightly) [22,23,24] on a 9-point hedonic scale. All experiments were performed in triplicate and compared with predictive values for model verification.

### 2.4. Determination of Physical Properties

The snack samples were determined for (i) water activity (a_w_) using a water activity measurement instrument (model ms1–1M, Novasina, Lachen, Switzerland) and (ii) color using a Colorflex EZ Spectrophotometer (HunterLab, Reston, VA, USA). The color was recorded as L* (lightness), a* (red–green), and b* (yellow–blue). (iii) Bulk density (BD) was determined as grams per cubic centimeter on a dry basis using a seed displacement method according to Chiu et al. [25]. BD was calculated using Equation (1) as follows:


(1) $$\mathrm{Bulk}\;\mathrm{density}\;(g/\mathrm{mL})\;=\frac{\mathrm{Weight}\;\mathrm{of}\;\mathrm{sample}\;(g)}{\mathrm{Volume}\;\mathrm{of}\;\mathrm{sample}\;(\mathrm{mL})}$$

(iv) A texture analyzer TA-XT plus (Stable Micro Systems, Godalming, Surrey, UK) and a 2-mm diameter aluminum cylinder probe (P/2) were used to measure the hardness. The maximum force applied was considered to be the hardness. Measurements were performed at a test speed of 2 mm/s, a post-test speed of 10 mm/s, and a test height of 5 mm. Ten replications were performed for each sample with a 50 kg load cell, and the average results were calculated [26].

### 2.5. Nutritional and Amino Acid Profiles Analysis

The proximate compositions of the *W. globosa* powder (WP), control snack, and developed *W. globosa* snack including moisture, ash, protein, fat and total dietary fiber were determined following the Association of Official Analytical Chemists (AOAC, 2019) [27]. Total carbohydrate was calculated by the subtraction of moisture, fat, protein, and ash contents from 100. Energy value was attained from the integration of total energy from carbohydrate, protein, and fat as 4, 4, and 9 kcal/g samples, respectively. The amino acid profile was constructed using high-performance liquid chromatography according to an in-house method TE-CH-372 adapted from the *Official Journal of the European Communities*, L257/16 [28]. Nutritional and amino acid profile analyses were determined by the testing laboratories of the Central Laboratory (Thailand) Co., Ltd., Bangkok, Thailand.

### 2.6. Sensory Evaluation

The snacks were tested to evaluate organoleptic attributes including appearance, color, odor, taste, texture, and overall acceptability [29] by 50 untrained panelists (ages 18–60 years old, no history of allergy to ingredients used). A 9-point hedonic scale rating 1 for dislike extremely, 5 for neither like nor dislike, and 9 for like extremely was utilized in this study. For each sample, panelists received a sample served in a bag (10 g) coded with a 3-digit random number to avoid bias. Panelists were provided with drinking water to clean their mouth between consecutive tastings.

### 2.7. Microbiological Quality

Total plate count, yeast and mold counts (YMCs), *Salmonella* spp., and *Staphylococcus aureus* counts were performed according to standard protocols of the *Bacteriological Analytical Manual* (BAM) [30]. The numbers of colonies appearing on the dilution plates were counted, averaged, and reported as colony forming units (CFUs)/g for total plate count and YMCs, while *Salmonella* spp. and Staphylococcus aureus were reported as CFUs/25 g.

### 2.8. Sample Extraction and Determination of Phytochemicals

The sample extraction was carried out in accordance with a prior study with slight modifications [31]. In brief, the fine powder of WP, control snack, and *W. globosa* snack were extracted with 70% (*v*/*v*) aqueous ethanol with solid-to-liquid ratio at 1:30 and shaken in a water bath shaker (Memmert GmBh, Eagle, WI, USA) for 2 h at 50 °C. The supernatants were collected by centrifugation at 3800× *g* for 10 min using a Hettich^®^ ROTINA 38R refrigerated centrifuge (Andreas Hettich GmbH, Tuttlingen, Germany). The extracts were stored at −20 °C until analysis.

Total phenolic contents (TPCs), total flavonoid contents (TFCs), and total tannin contents (TTCs) were determined with the well-establish protocols as previously detailed [32,33]. In brief, TPCs and TTCs were measured using Folin–Ciocalteu reagent and recorded at 765 and 700 nm, respectively. TPCs were presented as mg gallic acid equivalent (GAE)/g DW and TTCs were expressed as mg tannic acid equivalent (TAE)/100 g DW. TFCs in the extracts were measured using aluminium chloride colorimetric assay with AlCl_3_·6H_2_O reagent. The signals were recorded at 510 nm. The results were expressed as mg quercetin equivalent (QE)/g DW. All absorbances were detected using a SynergyTM HT 96-well UV-visible microplate reader and Gen 5 data analysis software (BioTek Instruments, Inc., Winooski, VT, USA).

### 2.9. Determination of Antioxidant Properties

Three antioxidant assays, including 2,2-diphenyl-1-picrylhydrazyl (DPPH) radical scavenging, ferric ion reducing antioxidant power (FRAP), and oxygen radical absorbance capacity (ORAC) assays were employed as formerly detailed [33]. The scavenging activities against DPPH radicals were measured by the end-point reaction at 520 nm. The reducing antioxidant power (Fe^3+^ to Fe^2+^) was also assayed by the end-point reaction at 600 nm, and inhibition of radicals induced by 2,2′-azobis(2-amidinopropane) dihydrochloride in the ORAC assay was kinetically measured at 485 nm excitation wavelength and 528 nm emission wavelength. The reactions were detected using the 96-well microplate reader. Trolox was used as a standard, and the results were reported as µmol Trolox equivalent (TE)/g DW.

### 2.10. Bacterial Reverse Mutation Assay (Ames Test)

*W. globosa* ethanolic extract (10–2000 µg/plate) were determined for its genotoxicity properties using the Organisation for Economic Co-operation and Development (OECD) guideline for testing of chemicals No. 471 ‘Bacterial Reverse Mutation Test’ [34]. *Salmonella typhimurium* bacteria including TA98, TA100, TA102, TA1535, and TA1537 were used as testing models in the absence or presence of metabolic activation with liver S9 extract (Sigma-Aldrich, St. Louis, MO, USA), to cover both direct and indirect-acting mutagens. Distilled water was used as a solvent control (negative control). The mutagenicity ratio (MR) was determined from the average of the revertant number divided by the average of negative control revertant number as previously reported [35].

### 2.11. Statistical Anylysis

The experimental design, regression, and graphical analysis of the generated data were performed using the software Design-Expert (Stat-Ease Inc., Minneapolis, MN, USA). Experiments were carried out in triplicate (*n* = 3) and reported as mean ± standard deviation (SD). The one-way analysis of variance (ANOVA) and Duncan’s multiple comparison test were used to examine the difference between samples in experiments and performed using SPSS version 18 (Statistical Package for the Social Sciences, SPSS Inc., Chicago, IL, USA). *p* < 0.05 was considered as significant difference.

## 3. Results

### 3.1. Effect of Different Ingredient Ratios on Snack Quality

#### 3.1.1. Physical Properties

The physical properties of ten *W. globosa* snacks including a_w_, color (L*, a*, b*), BD, and texture (hardness) are shown in Table 2. All snack samples exhibited a_w_ ranging from 0.09 to 0.23. When incorporating WP, the snack color L*, a*, and b* values varied from 34.05 to 40.62, −2.95 to −0.4, and 26.98 to 29.09, respectively. A higher proportion of WP resulted in a significantly greener color, while a higher proportion of glutinous rice (GF) gave a significantly lighter and more yellowish color. Incorporation of WP had a significant effect on the BD and hardness of the snack samples, ranging from 0.22 to 0.31 g/mL and from 1658 to 2448.54 g, respectively. High correlation between bulk density and hardness was demonstrated at higher proportions of WP.

#### 3.1.2. Nutritional Values

Table 3 shows the proximate analysis results (per 100 g DW) of ten *W. globosa* snack samples consisting of 404.24–439.36 kcal, 58.84–67.30 g carbohydrate, 8.33–13.93 g protein, 11.76–12.95 g fat, and 6.15–12.63 g dietary fiber. Nutritional values of all snack samples varied significantly, with incorporation of WP resulting in a significant increase in protein (25–55%) and dietary fiber (72–87%).

#### 3.1.3. Sensory Evaluation

Sensory evaluation is conducted to assess public liking of food products. A 9-point hedonic scale was used to determine many aspects of sensory assessment. The effects of WP incorporation on snack sensory scores are shown in Table 4. Average sensory scores of all snack samples ranged from 5.70–7.60 (like slightly to like very much), indicating that the products were satisfactory [22,23,24]. Results also showed that higher amounts of WP had a negative impact on texture and liking scores, while no change in product color was observed.

### 3.2. Optimization of Ingredients for W. globosa Snack Development

**Figure 1:**
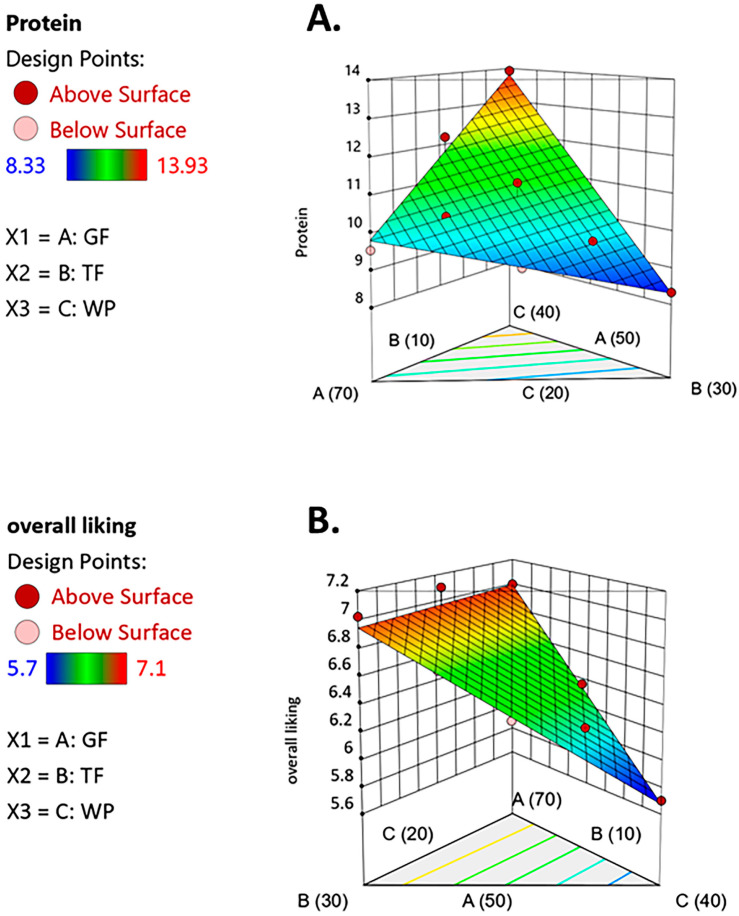
3D plots of protein (**A**) and overall liking (**B**); glutinous rice flour (GF, X_1_) = 50–70% *w*/*w*, tapioca flour (TF, X_2_) = 10–30% *w*/*w*, and *W. globosa* powder (WP, X_3_) = 20–40% *w*/*w*.

RSM was used to study the relationships between the ratios of the independent variables (GF (X_1_), TF (X_2_), and WP (X_3_)) and the response variables (protein (Y_1_) and overall liking (Y_2_)) using a regression model, as shown in Table 5. The coefficient of determination (R^2^) values of the protein and overall liking response variables were 0.97 and 0.96, respectively. Higher R^2^ values than 89% indicated the fitness of the polynomial models used to explain the effect of the variables on the responses. Significant difference was observed in the *p* values, suggesting that the independent variables in the model had a relationship with the dependent variable at a significance level of 95%. Figure 1 shows a 3D plot of protein and overall liking. The optimal formulation was created using the desirability function approach. The criteria chosen for optimizing the independent variables in the snack formulation were protein >10 g/100 g (10% Thai RDI) [20,21] and overall liking score more than 6 (like slightly) on a 9-point hedonic scale [22,23,24]. The optimal solution was obtained at 64% *w*/*w* GF, 10% *w*/*w* TF, and 26% *w*/*w* WP, with a desirability liking score of 1.00.

The optimal levels of independent variables predicted by the models were compared with the actual values obtained from the optimized snack formulation to verify the model predictions. The predicted results of optimized snack formulation were protein at 10.99 g/100 g DW and overall liking score of 6.56 (light slightly to like moderately). All optimized snack formulation experiments were performed in triplicate and actual results were given as protein 10.86 ± 0.07 g/100 g DW and overall liking score 6.82 ± 0.90 (light slightly to like moderately). No significant differences were found between the experimental results and the predicted values (*p* ≥ 0.05), confirming the adequacy of the developed models for identifying the relationships between the independent and response variables.

### 3.3. Comparison of the Control and Developed W. globosa Snack Quality

Using the optimized ingredients in Section 3.2, the developed *W. globosa* snack was compared with WP and the control snack with the active ingredients indicated in Table 6 regarding their nutritional values, amino acid profiles, sensory evaluation, microbiological quality, phytochemicals, and antioxidant activities. In addition, mutagenicity potential of WP was also investigated.

#### 3.3.1. Nutritional Values and Amino Acid Profiles

Table 7 shows the nutritional values (g/100 g DW) of *W. globosa* powder (WP), control snacks (0% WP), and *W. globosa* snacks (26% WP). The major component in WP (100 g DW) was carbohydrate (52.59 g), with dietary fiber (36.52 g), protein (31.50 g), fat (5.18 g), and ash (10.73 g). Therefore, WP addition improved the nutrient value of the snack. Proximate analysis of the control and *W. globosa* snacks showed that addition of WP increased protein and dietary fiber by 51% and 83%, respectively. No significant differences in energy, fat, or ash were recorded, while the *W. globosa* snack contained significantly lower carbohydrate than the control snack.

Amino acid compositions of WP, the control snack, and the developed *W. globosa* snack are shown in Table 8. The nutritional quality of protein depends on its essential amino acids (EAAs). Results revealed that WP and the developed *W. globosa* snack contained nine EAAs (histidine, isoleucine, leucine, lysine, methionine, phenylalanine, threonine, tryptophan, and valine), while methionine was not found in the control snack. The total essential amino acid (TEAA) and total non-essential amino acid (TNEAA) ratios of WP, the control, and the *W. globosa* snack were 0.68%, 0.58%, and 0.64%, respectively. The major amino acids in WP were aspartic acid, glutamic acid, alanine, and leucine at 3275, 3283, 2681, and 2367 mg/100 g, respectively, giving a trend similar to the *W. globosa* snack. Addition of WP increased the amino acid composition in the *W. globosa* snack more than in the control snack. The *W. globosa* snack also had significantly higher hydrophilic amino acids (3-fold higher), hydrophobic amino acids (2-fold higher), acidic amino acids (3-fold higher), and basic amino acids (2-fold higher) than the control snack.

#### 3.3.2. Sensory Evaluation and Microbiological Quality

**Figure 2:**
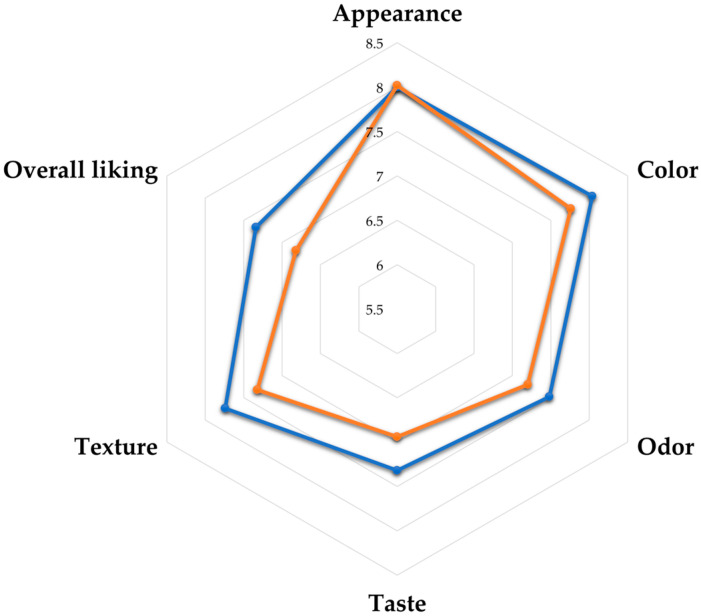
Sensory properties of control snack (blue color) and developed *W. globosa* snack (orange color).

Sensory evaluations of the control and *W. globosa* snacks were performed by untrained panelists (*n* = 50) using a 9-point hedonic scale, with results shown in Figure 2. No significant differences were recorded in appearance scores between the control and developed *W. globosa* snacks, while significantly higher scores for color, odor, taste, texture, and overall liking were observed in the control snack. However, both the control and *W. globosa* snacks were accepted by the panelists, with all attributes scores higher than 6 (like slightly) [22,23,24]. The microbiological quality of the *W. globosa* snack was analyzed according to the Thai community product standard for crispy snacks. This standard states that total plate counts, YMCs, and *Staphylococcus aureus* must be less than 1 × 10^6^, 100, and 10 CFUs/1 g of sample, respectively, while *Salmonella* spp. must not be found in a 25 g sample [36]. Results showed that total plate counts, YMCs, and *S. aureus* content of the *W. globosa* snack were in line with the standard (<10 CFUs/g) and no *Salmonella* spp. were detected in the high protein snack (25 g).

### 3.4. Phytochemicals and Antioxidant Activities of W. globosa Snack

The phytochemicals in *W. globosa* have known antioxidant activities [14]. Thus, phytochemicals (TPCs, TFCs, and TTCs) and antioxidant properties (DPPH radical scavenging, FRAP, and ORAC activities) were determined in order to investigate the health benefits of the developed *W. globosa* snack. Table 9 reveals that WP had TPCs, TFCs, and TTCs at 11.67 mg of GAE/g DW, 12.51 mg of QE/g DW, and 32.31 mg of TAE/g DW, respectively, while these values decreased by four- to five-fold in the *W. globosa* snack and were barely present in the control snack. The antioxidant activities correlated with the amount of phytochemicals. WP exhibited the highest antioxidant activities in all three assays, followed by the *W. globosa* snack. Results implied that addition of WP increased the phytochemicals and antioxidant activities in the developed *W. globosa* snack.

### 3.5. Evaluation of Mutagenicity Potential of W. globosa Powder (WP) Ethanolic Extract

*W. globosa* is a novel ingredient in functional food but has been used as a food source for a long time. Genotoxicity testing is an important requirement for functional food development. Therefore, WP was subjected to genotoxicity testing (Ames test) following the OECD guidelines. Table 10 shows the mutagenicity effects of WP ethanolic extract on *S. typhimurium* strains without rat liver S9, while Table 11 shows the mutagenicity effects of WP ethanolic extract on *S. typhimurium* strains with rat liver S9. The experiment used rat liver S9 extract to determine whether WP was a direct or indirect mutagen. Compared with the positive controls, both Table 10 and Table 11 show that the number of revertant colonies remained the same in all five bacterial strains treated with WP, as in the negative control, even if the concentration was extremely high (2000 µg/plate). This result indicated that WP did not induce DNA mutations and was genome-safe, reflecting the genome safety of *W. globosa* snacks.

## 4. Discussion

Nowadays, people are more concerned about their health and desire healthy and nutritious snacks. Demand for snack products is increasing with changing personal eating habits. Normally, high-energy-density food snacks are made from rice and maize, with low quality of protein due to lack of essential amino acids. Blending nutrient-rich ingredients in snacks, such as bean [37,38], can assist in ameliorating the risk of noncommunicable diseases (NCDs) such as obesity, diabetes, and cardiovascular disease. *W. globosa* is a natural food source which contains high protein [39,40]. Many researchers have reported on the benefits of *W. globosa*. These include postprandial glycemic effects [41] and may play a role in the regression of visceral adiposity [42]. Therefore, this study optimized the ingredients to develop a *W. globosa* high-protein snack using RSM with a mixture design based on sensory (overall liking score) and nutritional (protein content) parameters. The results highlight the potential of using *W. globosa* to improve the nutritional contents of snack products.

RSM with mixture design was used to develop *W. globosa* snacks, and the physical properties, nutritional values, and sensory attributes of different proportions of ingredients (glutinous rice flour, tapioca flour, and *W. globosa* powder) were analyzed. Water activity (a_w_) of all snack formulations was low (0.09–0.23), and almost all bacteria, yeast, and mold could not survive, thereby extending product shelf life [43]. Higher proportions of WP resulted in significantly greener snack products due to the intense green pigment of chlorophylls [44,45]. WP had a marked influence on bulk density (BD) and hardness, with maximum values recorded at 40% WP content. This finding concurred with several researchers [46,47,48] who found that addition of high fiber and protein increased the density and hardness of snacks. Increased fiber and protein contents encouraged interactions between polysaccharides and proteins, inhibiting starch matrix puffing during the heating process [46,47,48]. Nutrient values of all snack formulations showed increased protein and fiber when WP percentage increased. The highest protein and fiber contents were found as expected in the snack with 40% WP, because WP is a good source of protein and fiber [14,39,40,49]. Sensory evaluation is often used to determine public acceptance of a product. Results revealed that higher WP reduced texture and overall liking scores, with harder texture of the snack after WP addition due to increased fiber content. The fiber inhibited starch swelling and increased cell wall thickness, thereby reducing porosity [50]. The sensory score for snack color was 7 (like moderately) and the greenish color did not adversely affect sensory perception. Gámbaro et al. (2006), Giménez et al. (2007) and Giménez et al. (2008) considered the minimum acceptability limit for consumers liking a product as 6 (like slightly) [22,23,24]. Therefore, using this criterion, all snack formulations were accepted by the panelists. Based on the criteria chosen for optimized levels of the independent variables for snack formulation including protein >10 g/100 g (10% of Thai RDI) [20,21] and overall liking score of more than 6 (like slightly) on a 9-point hedonic scale, the combination of 64% GF, 10% TF, and 26% WP gave the highest desirability values (1.00). Ruiz-Armenta (2018) considered a desirability value of 0.60 as acceptable [51]. Thus, this formulation was used to develop a *W. globosa* snack with high protein and dietary fiber. The validation test confirmed that the model adequately predicted the optimal high-protein snack formulation with WP.

Nutritional compositions of WP, the control snack (0% WP), and the *W. globosa* snack (26% WP) were analyzed. Results showed that carbohydrate, protein, fat, ash, and fiber contents of WP concurred with other studies [14,39,40,49]. Based on Thai RDIs, 100 g of WP provides protein and fiber at up to 63% and 146%, respectively [20,21]. Protein is a macronutrient that is required to maintain body growth and development, while fiber is associated with fewer metabolic diseases and plays an important role in intestinal health [52]. Therefore, WP shows promise as a good alternative protein source to improve the nutrients of the product. One serving size (30 g) of *W. globosa* snack contains 3.86 g protein and 2.90 g fiber or 12.86% and 19.36% Thai RDI, respectively [20,21]. This product could be marketed as high protein and fiber, with protein and fiber contents higher than 10 g and 6 g/100 g of product, respectively [20,21]. The *W. globosa* snack showed significantly decreased carbohydrate content due to replacement of GF and TF with WP. Similar results were reported for starch content decrease in bean-based products [53,54,55]. Amino acid profiles showed that WP high-protein snacks contained all nine indispensable amino acids [40], with significantly higher amino acid compositions than the control snack. WP contains aquatic proteins and fiber-rich plant material [14,39,40,49], with high leucine as the most powerful anabolic agent [56]. Many studies have reported the positive effect of leucine on protein synthesis [57,58]. WP snacks could be eaten as a functional food because the hydrophobic amino acids act as antioxidants by increasing the solubility of peptides in lipids, which facilitates better interaction with free radicals [59,60]. The sensory evaluation revealed that the developed *W. globosa* snack was accepted by the panelists, with sensory attribute scores above 6 [22,23,24]. The microorganism quality also showed that the high-protein snack was safe for consumption according to the Thai community product standard for crispy snacks [36].

Duckweeds contain macronutrients and they are also rich in phytochemicals, which are associated with a variety of health benefits including antioxidant properties, anti-cancer, anti-obesity, anti-diabetes, and anti-aging properties [16]. In this study, the WP ethanolic extract contained TPCs, TFCs, and TTCs at 1.16%, 1.25%, and 3.23% (Table 9). Somdee et al. reported a similar range of TPCs in *W. globosa* at 1.24% [61], while another study on *Lemna minor* (common duckweed) showed TPCs below 3% [62], concurring with our results. Duckweeds have significantly greater flavonoid contents (>2%) than the vast majority of plants (0.5–1.5%) [9]. We reported TFCs of *W. globosa* at 1.25%, while Somdee et al. reported TFCs at 0.25% [61], and Zhao et al. reported TFCs at 5.85% [63]. These results suggest some variations in TFCs. Many variables can impact the quantities of phytochemicals in plants, such as species, growing conditions, and location. Thus, further applications of duckweeds as functional foods must consider the standardization of bioactive compounds. This study did not cover phytochemical identification, but previous studies recorded several phytochemical compounds in *W. globosa* including ferulic acid, luteolin 7-*O*-β-d-glucoside, kaempferol, β-sitosterol, and stigmasterol. These compounds have known human health benefits, as mentioned earlier. The WP extract and *W. globosa* snacks showed antioxidant properties, especially when measured by ORAC assay (Table 8). Addition of WP to snacks clearly enhanced the antioxidant value. The ORAC assay quenches free radicals by hydrogen atom transfer (HAT) instead of the single electron transfer (SET) mechanism [64], and is more relevant to organisms compared with other readouts [65]. TTCs were also recorded in WP, in line with our study. *W. arrhiza*, a close species to *W. globosa*, had tannins at 9.83 mg/DW [31], three-fold lower than our report. Tannins act as health-promoting and anti-nutritional compounds because they inhibit digestive enzymes and bind to nutrients, eventually leading to poor absorption of some vitamins and minerals [66]. Fortunately, tannins did not appear to contribute to the bioavailability of EAAs in *W. globosa* that was comparable to soft cheese and peas, as determined in male subjects in a randomized controlled trial [40]. Consumption of *W. globosa* could reduce blood glucose concentration and next-morning fasting glucose levels [41]. Future studies should investigate the postprandial glycemic response of our developed *W. globosa* snacks.

## 5. Conclusions

*Wolffia globosa* possesses high protein, dietary fiber, and phytochemicals, rendering it a potential novel food source to ameliorate malnutrition through development of a functional food. This study formulated a *W. globosa*-based snack using RSM with mixture design. The developed products exhibited high protein, EAAs, dietary fiber, phytochemicals, and antioxidant activities and were devoid of mutagenic potential. Addition of WP as a healthy and nutritious ingredient shows promise in the snack industry.

## Figures and Tables

**Table 1:** Experimental design layout by response surface methodology (RSM).

Run	Actual Variables		
	X_1_ (% *w*/*w*)	X_2_ (% *w*/*w*)	X_3_ (% *w*/*w*)
1	50	10	40
2	70	10	20
3	50	30	20
4	50	20	30
5	60	10	30
6	60	20	20
7	56.67	16.66	26.67
8	53.34	13.33	33.33
9	63.34	13.33	23.33
10	53.34	23.33	23.33

X_1_: glutinous rice flour (% *w*/*w*), X_2_: tapioca flour (% *w*/*w*), X_3_: *W. globosa* powder (% *w*/*w*).

**Table 2:** Physical properties of ten developed *W. globosa* snacks including water activity (a_w_), color, bulk density (BD), and texture (hardness).

Formulations	a_w_	Color			BD (g/mL)	Hardness (g)
		L*	a*	b*		
1	0.09 ± 0.00 ^g^	34.05 ± 0.05 ^h^	−0.49 ± 0.00 ^a^	28.08 ± 0.11 ^c^	0.31 ± 0.00 ^a^	2448.54 ± 238.11 ^a^
2	0.23 ± 0.00 ^a^	38.70 ± 0.06 ^d^	−1.57 ± 0.04 ^d^	28.93 ± 0.09 ^ab^	0.29 ± 0.00 ^b^	1950.84 ± 495.78 ^bc^
3	0.21 ± 0.01 ^b^	40.39 ± 0.01 ^b^	−2.95 ± 0.02 ^f^	29.09 ± 0.05 ^a^	0.26 ± 0.01 ^c^	1658.63 ± 246.32 ^c^
4	0.19 ± 0.01 ^c^	39.02 ± 0.03 ^c^	−2.69 ± 0.01 ^ef^	28.91 ± 0.07 ^bc^	0.22 ± 0.01 ^f^	2183.07 ± 170.56 ^ab^
5	0.20 ± 0.00 ^b^	40.62 ± 0.01 ^a^	−2.51 ± 0.00 ^e^	28.59 ± 0.15 ^b^	0.22 ± 0.00 ^f^	1943.36 ± 507.53 ^bc^
6	0.23 ± 0.00 ^a^	40.27 ± 0.15 ^b^	−1.11 ± 0.55 ^bc^	26.98 ± 0.60 ^d^	0.24 ± 0.01 ^d^	1950.68 ± 380.63 ^bc^
7	0.17 ± 0.00 ^e^	38.60 ± 0.00 ^d^	−1.33 ± 0.02 ^cd^	27.44 ± 0.04 ^d^	0.23 ± 0.01 ^def^	1868.12 ± 472.86 ^bc^
8	0.10 ± 0.00 ^f^	37.40 ± 0.07 ^g^	−0.85 ± 0.04 ^ab^	27.34 ± 0.19 ^d^	0.22 ± 0.00 ^ef^	1909.48 ± 373.28 ^bc^
9	0.16 ± 0.00 ^e^	37.89 ± 0.16 ^f^	−0.93 ± 0.03 ^b^	27.00 ± 0.14 ^d^	0.23 ± 0.01 ^de^	2020.85 ± 301.14 ^bc^
10	0.17 ± 0.00 ^d^	38.40 ± 0.08 ^e^	−0.79 ± 0.03 ^ab^	27.34 ± 0.11 ^d^	0.23 ± 0.01 ^de^	2010.21 ± 492.46 ^bc^

All data are shown as the mean ± standard deviation (SD) of triplicate determination (*n* = 3). Different lowercase letters denote significant differences in a_w_, color, BD, or hardness at *p* < 0.05 in different snack formulations using one-way ANOVA, followed by Duncan’s multiple comparison test. The ratio of glutinous rice flour: tapioca flour: *W. globosa* powder in each formulation is shown in Table 1. Color is expressed in CIELAB units (L* represents dark (0) to white (100) colors, a* represents green (−) to red (+) colors, and b* represents blue (−) to yellow (+) colors).

**Table 3:** Proximate compositions of ten *W. globosa* snacks (g/100 g dry weight).

Formulations	Energy (kcal)	Carbohydrate (g)	Protein (g)	Fat (g)	Dietary Fiber (g)
1	428.06 ± 3.41 ^b^	59.34 ± 0.47 ^f^	13.93 ± 0.11 ^a^	12.95 ± 0.10 ^a^	12.63 ± 0.10 ^a^
2	420.45 ± 2.69 ^c^	66.50 ± 0.43 ^bc^	9.53 ± 0.06 ^g^	11.92 ± 0.08 ^e^	6.21 ± 0.04 ^hi^
3	415.11 ± 2.32 ^d^	66.74 ± 0.37 ^abc^	8.33 ± 0.05 ^i^	11.76 ± 0.07 ^f^	6.15 ± 0.03 ^i^
4	412.64 ± 1.35 ^d^	61.67 ± 0.20 ^e^	10.86 ± 0.04 ^e^	12.09 ± 0.04 ^d^	9.15 ± 0.03 ^d^
5	442.25 ± 0.70 ^a^	65.63 ± 0.10 ^d^	12.21 ± 0.02 ^b^	12.96 ± 0.02 ^a^	9.79 ± 0.02 ^c^
6	422.01 ± 2.91 ^c^	67.30 ± 0.46 ^a^	9.02 ± 0.06 ^h^	11.96 ± 0.08 ^e^	6.24 ± 0.04 ^h^
7	439.36 ± 1.84 ^a^	67.00 ± 0.28 ^ab^	11.03 ± 0.05 ^d^	12.73 ± 0.05 ^b^	8.66 ± 0.04 ^e^
8	404.24 ± 1.26 ^e^	58.84 ± 0.18 ^f^	11.65 ± 0.04 ^c^	11.97 ± 0.04 ^e^	9.95 ± 0.03 ^b^
9	429.85 ± 0.81 ^b^	66.78 ± 0.13 ^abc^	10.26 ± 0.02 ^f^	12.32 ± 0.02 ^c^	7.41 ± 0.01 ^f^
10	423.09 ± 2.32 ^c^	66.27 ± 0.36 ^c^	9.55 ± 0.05 ^g^	12.13 ± 0.07 ^d^	7.30 ± 0.04 ^g^

All data are shown as the mean ± standard deviation (SD) of triplicate determination (*n* = 3). Different lowercase letters denote significant differences in the contents of the same proximate composition at *p* < 0.05 in different snack formulations, using one-way ANOVA followed by Duncan’s multiple comparison test. The ratio of glutinous rice flour: tapioca flour: *W. globosa* powder in each formulation is shown in Table 1. Nutrient values were calculated using INMUCAL-Nutrients V.4.0.

**Table 4:** Sensory evaluation of ten *W. globosa* snacks.

Formulations	Appearance	Color ^ns^	Odor	Taste	Texture	Overall Liking
1	7.34 ± 0.88 ^abc^	7.00 ± 1.06	6.26 ± 1.52 ^c^	5.78 ± 1.63 ^c^	6.20 ± 1.47 ^d^	5.70 ± 1.72 ^d^
2	7.56 ± 0.98 ^a^	7.44 ± 1.00	7.16 ± 1.08 ^a^	7.00 ± 1.31 ^a^	7.26 ± 1.02 ^a^	7.00 ± 1.22 ^ab^
3	7.62 ± 0.93 ^a^	7.32 ± 0.97	7.14 ± 0.98 ^a^	6.90 ± 1.15 ^ab^	7.20 ± 1.02 ^ab^	7.02 ± 1.05 ^ab^
4	7.08 ± 0.93 ^c^	7.14 ± 0.98	6.58 ± 1.04 ^bc^	6.30 ± 1.14 ^bcd^	6.54 ± 1.17 ^cd^	6.28 ± 1.10 ^c^
5	7.12 ± 1.19 ^bc^	7.12 ± 1.12	6.50 ± 1.19 ^bc^	6.24 ± 1.19 ^cd^	6.68 ± 1.21 ^bcd^	6.36 ± 1.13 ^c^
6	7.52 ± 0.85 ^ab^	7.36 ± 0.79	7.16 ± 0.86 ^a^	6.84 ± 1.05 ^abc^	7.36 ± 0.84 ^a^	7.10 ± 0.88 ^a^
7	7.22 ± 1.03 ^abc^	7.18 ± 1.05	6.70 ± 1.24 ^abc^	6.44 ± 1.51 ^abc^	6.84 ± 1.47 ^abc^	6.46 ± 1.46 ^bc^
8	7.30 ± 1.06 ^abc^	7.14 ± 1.30	6.56 ± 1.34 ^bc^	6.24 ± 1.42 ^cd^	6.68 ± 1.25 ^bcd^	6.16 ± 1.57 ^cd^
9	7.50 ± 0.78 ^abc^	7.40 ± 0.80	6.96 ± 1.11 ^ab^	6.50 ± 1.45 ^abc^	7.12 ± 1.16 ^ab^	6.70 ± 1.06 ^abc^
10	7.48 ± 0.72 ^abc^	7.36 ± 0.91	6.68 ± 1.26 ^abc^	6.40 ± 1.50 ^abc^	7.02 ± 1.14 ^abc^	6.58 ± 1.39 ^abc^

All data are shown as the mean ± standard deviation (SD) according to 50 untrained panelists (*n* = 50). Different lowercase letters denote significant differences in values at *p* < 0.05, while ‘ns’ denotes no significant differences in values at *p* ≥ 0.05 for the same sensory attributes in different snack formulations, using one-way ANOVA followed by Duncan’s multiple comparison test. The ratio of glutinous rice flour: tapioca flour: *W. globosa* powder in each formulation is shown in Table 1.

**Table 5:** Predictive regression models for protein and overall liking of an optimized *W. globosa* snack.

Response Variable	Predictive Model	R^2^	*p*-Value
Protein	Y_1_ = 0.065(X_1_) − 0.009(X_2_) + 0.266(X_3_)	0.97	<0.05
Overall liking	Y_2_ = 0.083(X_1_) + 0.081(X_2_) + 0.017(X_3_)	0.96	<0.05

**Table 6:** The active ingredients of *W. globosa* powder (WP), control snack, and developed *W. globosa* snack.

Ingredients (% *w*/*w*)	*W. globosa* Powder	Control Snack	*W. globosa* Snack
Glutinous rice flour	0	70	64
Tapioca flour	0	30	10
*W. globosa* powder	100	0	26

**Table 7:** Nutritional values of *W. globosa* powder (WP), control snack, and developed *W. globosa* snack (per 100 g dry weight).

Nutritional Values	*W. globosa* Powder	Control Snack	*W. globosa* Snack
Energy (kcal)	383.03 ± 0.71 ^b^	455.84 ± 5.51 ^a^	457.58 ± 5.35 ^a^
Carbohydrate (g)	52.59 ± 0.16 ^c^	78.49 ± 1.55 ^a^	71.50 ± 0.75 ^b^
Fat (g)	5.18 ± 0.01 ^b^	13.08 ± 0.16 ^a^	13.35 ± 0.08 ^a^
Protein (g)	31.50 ± 0.31 ^a^	6.28 ± 0.18 ^c^	12.86 ± 0.76 ^b^
Dietary fiber (g)	36.52 ± 0.21 ^a^	1.69 ± 0.01 ^c^	9.97 ± 0.07 ^b^
Ash (g)	10.73 ± 0.12 ^a^	1.72 ± 0.59 ^b^	2.79 ± 0.64 ^b^

All data are shown as the mean ± standard deviation (SD) of triplicate determination (*n* = 3). Different lowercase letters denote significant differences in contents of the same proximate composition at *p* < 0.05 in different samples, using one-way ANOVA followed by Duncan’s multiple comparison test. All active ingredients of the snacks are shown in Table 6.

**Table 8:** Amino acid profiles of WP, control snack, and *W. globosa* snack (mg/100 g).

Amino Acid Profiles	*W. globosa* Powder	Control Snack	*W. globosa* Snack
Essential amino acids			
Leucine	2367.97 ± 2.28 ^a^	418.52 ± 1.39 ^c^	961.30 ± 1.30 ^b^
Lysine	1672.86 ± 3.39 ^a^	113.16 ± 0.52 ^c^	511.42 ± 0.08 ^b^
Isoleucine	1091.63 ± 0.92 ^a^	191.06 ± 0.26 ^c^	437.13 ± 0.40 ^b^
Histidine	539.18 ± 1.34 ^a^	116.97 ± 0.14 ^c^	256.73 ± 0.54 ^b^
Tryptophan	335.29 ± 0.74 ^a^	148.47 ± 0.73 ^c^	162.04 ± 0.22 ^b^
Valine	1681.18 ± 1.32 ^a^	285.52 ± 1.27 ^c^	691.22 ± 0.49 ^b^
Methionine	254.75 ± 1.80 ^a^	ND	199.02 ± 0.19 ^b^
Phenylalanine	1623.17 ± 3.15 ^a^	261.86 ± 0.56 ^c^	622.99 ± 0.30 ^b^
Threonine	1178.07 ± 2.77 ^a^	198.15 ± 0.43 ^c^	442.85 ± 0.55 ^b^
TEAA	10,743.08	1733.69	4284.68
Nonessential amino acids			
Tyrosine	963.05 ± 2.79 ^a^	248.93 ± 1.34 ^c^	341.94 ± 0.10 ^b^
Cystine	ND	ND	ND
Alanine	2681.18 ± 2.26 ^a^	282.89 ± 0.96 ^c^	1000.18 ± 1.82 ^b^
Glutamic acid	3283.25 ± 3.06 ^a^	960.18 ± 1.18 ^c^	1668.82 ± 1.68 ^b^
Glycine	1476.53 ± 2.60 ^a^	207.82 ± 1.17 ^c^	575.78 ± 0.66 ^b^
Arginine	1692.51 ± 3.73 ^a^	368.75 ± 0.61 ^c^	754.03 ± 0.06 ^b^
Aspartic acid	3275.10 ± 1.62 ^a^	439.46 ± 0.80 ^c^	1290.45 ± 0.84 ^b^
Serine	1250.41 ± 2.76 ^a^	260.75 ± 1.36 ^c^	555.92 ± 0.32 ^b^
Proline	1267.16 ± 2.42 ^a^	228.41 ± 1.02 ^c^	478.92 ± 0.61 ^b^
TNEAA	15,889.16	2997.18	6666.02
Hydrophilic amino acids	4868.05	915.64	1916.48
Hydrophobic amino acids	10,966.02	1668.25	4390.74
Acidic amino acids	3904.55	1399.63	2959.27
Basic amino acids	6558.34	598.88	1522.18

All data are shown as the mean ± standard deviation (SD) of triplicate determination (*n* = 3). Different lowercase letters denote significant differences in contents of the same proximate composition at *p* < 0.05 in different samples, using one-way ANOVA followed by Duncan’s multiple comparison test. All active ingredients of the snacks are shown in Table 6. TEAA: total essential amino acids = leucine + lysine + isoleucine + phenylalanine + tryptophan + valine + methionine + histidine + threonine; TNEAA: total non-essential amino acids = tyrosine + cystine + alanine + glutamic acid + glycine + aspartic acid + serine + proline + arginine; hydrophobic amino acids = methionine + alanine + valine + leucine + isoleucine + proline + phenylalanine; hydrophilic amino acids = glycine + tyrosine + serine + threonine + cysteine; basic amino acids = lysine + histidine + arginine; acidic amino acids = glutamic acid + aspartic acid.

**Table 9:** Phytochemicals and antioxidant properties of *W. globosa* powder (WP), control snack, and *W. globosa* snack.

Samples	TPCs (mg of GAE/g DW)	TFCs (mg of QE/g DW)	TTCs (mg of TAE/g DW)	Antioxidant Activities (µmol of TE/g DW)		
				DPPH Radical Scavenging Assay	FRAP Assay	ORAC Assay
WP extract	11.67 ± 0.11 ^a^	12.51 ± 0.38 ^a^	32.31 ± 1.00 ^a^	55.01 ± 4.26 ^a^	63.19 ± 4.03 ^a^	397.52 ± 27.20 ^a^
Control snack	ND	ND	1.41 ± 0.17 ^c^	2.50 ± 0.21 ^c^	ND	0.83 ± 0.04 ^b^
*W. globosa* snack	2.94 ± 0.04 ^b^	3.11 ± 0.08 ^b^	6.97 ± 0.39 ^b^	8.95 ± 0.73 ^b^	15.94 ± 2.05 ^b^	191.80 ± 14.41 ^c^

All data are represented as mean ± standard deviation (SD) of triplicate experiments (*n* = 3). The lowercase letters specify significantly different contents in the same column at *p* < 0.05 using one-way ANOVA and Duncan’s multiple comparison test. All active ingredients in the extract and snacks are shown in Table 6. TPCs: total phenolic contents; TFCs: total flavonoid contents; TTCs: total tannin contents; DPPH: 2,2-diphenyl-1-picrylhydrazyl; FRAP: ferric ion reducing antioxidant power; ORAC: oxygen radical absorbance capacity; GAE; gallic acid equivalent; QE: quercetin equivalent; TAE: tannic acid equivalent; TE: Trolox equivalent; DW: dry weight; ND: not detected.

**Table 10:** Mutagenicity effects of *W. globosa* ethanolic extract on five *S. typhimurium* strains without rat liver S9 extract (-S9).

Doses (μg/Plate)	TA98		TA100		TA102		TA1535		TA1537	
	Revertant Colonies	MR	Revertant Colonies	MR	Revertant Colonies	MR	Revertant Colonies	MR	Revertant Colonies	MR
Neg	82.83 ± 2.19	1.00 (–)	69.83 ± 2.73	1.00 (–)	353.67 ± 6.75	1.00 (–)	9.83 ± 1.07	1.00 (–)	11.17 ± 1.07	1.00 (–)
10	84.83 ± 2.79	1.02 (–)	67.17 ± 2.19	0.96 (–)	356.50 ± 4.99	1.01 (–)	10.00 ± 1.00	1.02 (–)	10.00 ± 0.82	0.90 (–)
100	81.50 ± 4.46	0.98 (–)	67.50 ± 1.38	0.97 (–)	357.33 ± 5.22	1.01 (–)	10.67 ± 1.11	1.08 (–)	10.33 ± 0.94	0.93 (–)
500	82.67 ± 2.29	1.00 (–)	69.50 ± 1.89	1.00 (–)	362.33 ± 7.32	1.02 (–)	11.00 ± 0.82	1.12 (–)	9.83 ± 0.69	0.88 (–)
1000	83.33 ± 3.45	1.01 (–)	68.83 ± 3.02	0.99 (–)	358.83 ± 6.39	1.01 (–)	9.17 ± 0.69	0.93 (–)	10.83 ± 0.69	0.97 (–)
2000	84.67 ± 2.87	1.02 (–)	68.50 ± 1.98	0.98 (–)	364.33 ± 5.44	1.03 (–)	10.17 ± 1.07	1.03 (–)	11.00 ± 0.82	0.99 (–)
4-NQO	1071.33 ± 27.94	12.93 (+)								
NaN_3_			1147.33 ± 13.74	16.43 (+)			260.50 ± 8.67	24.49 (+)		
MMC					1060.00 ± 24.00	3.00 (+)				
9-AA									742.67 ± 29.09	66.51 (+)

All data are shown as mean ± standard deviation (SD) of triplicate experiments (*n* = 3). Negative control (Neg) is distilled water used as a solvent control. MR: mutagenicity ratio; positive control: 4-NQO: 4-nitroquinoline-1-oxide; NaN_3_: sodium azide; MMC: mitomycin C; 9-AA: 9-aminoacridine; (–): indicates the mutagenicity ratio (MR) is ≤1; (+): indicates the mutagenicity ratio (MR) is ≥2.

**Table 11:** Mutagenicity effects of *W. globosa* ethanolic extract on five *S. typhimurium* strains with rat liver S9 extract (+S9).

Doses (μg/Plate)	TA98		TA100		TA102		TA1535		TA1537	
	Revertant Colonies	MR	Revertant Colonies	MR	Revertant Colonies	MR	Revertant Colonies	MR	Revertant Colonies	MR
Neg	87.00 ± 3.56	1.00 (–)	79.33 ± 3.99	1.00 (–)	363.17 ± 5.24	1.00 (–)	9.50 ± 0.76	1.00 (–)	10.50 ± 1.26	1.00 (–)
10	85.17 ± 3.13	0.98 (–)	73.50 ± 2.43	0.93 (–)	362.83 ± 4.52	1.00 (–)	10.50 ± 1.26	1.11 (–)	10.33 ± 0.94	0.98 (–)
100	87.00 ± 4.00	1.00 (–)	74.67 ± 3.14	0.94 (–)	360.50 ± 4.96	0.99 (–)	9.50 ± 0.96	1.00 (–)	10.00 ± 0.82	0.95 (–)
500	88.50 ± 3.95	1.02 (–)	74.00 ± 3.56	0.93 (–)	362.33 ± 6.18	1.00 (–)	10.67 ± 0.94	1.12 (–)	10.67 ± 1.11	1.02 (–)
1000	90.33 ± 3.45	1.04 (–)	73.83 ± 4.37	0.93 (–)	360.17 ± 5.34	0.99 (–)	10.33 ± 1.60	1.09 (–)	10.00 ± 1.00	0.95 (–)
2000	89.50 ± 2.22	1.03 (–)	79.00 ± 3.96	1.00 (–)	358.67 ± 5.31	0.99 (–)	9.33 ± 0.47	0.98 (–)	9.50 ± 0.76	0.90 (–)
2-AA	1125.33 ± 24.29	12.93 (+)	1018.67 ± 48.20	11.73 (+)	1140.00 ± 49.48	3.14 (+)	370.33 ± 9.03	38.98 (+)	199.33 ± 8.94	18.98 (+)

All data are shown as mean ± standard deviation (SD) of triplicate experiments (*n* = 3). Negative control (Neg) is distilled water used as a solvent control. MR: mutagenicity ratio; 2-AA: 2-aminoanthracen; (–): indicates the mutagenicity ratio (MR) is ≤1; (+): indicates the mutagenicity ratio (MR) is ≥2.

## Data Availability

The datasets generated for this study are available on reasonable request to the corresponding author.
